# CaMK4 overexpression in polycystic kidney disease promotes mTOR-mediated cell proliferation

**DOI:** 10.1093/jmcb/mjac050

**Published:** 2022-08-24

**Authors:** Yan Zhang, Emily A Daniel, July Metcalf, Yuqiao Dai, Gail A Reif, Darren P Wallace

**Affiliations:** Department of Internal Medicine, University of Kansas Medical Center, Kansas City, KS 66160-3018, USA; Jared Grantham Kidney Institute, University of Kansas Medical Center, Kansas City, KS 66160-3018, USA; Department of Internal Medicine, University of Kansas Medical Center, Kansas City, KS 66160-3018, USA; Jared Grantham Kidney Institute, University of Kansas Medical Center, Kansas City, KS 66160-3018, USA; Department of Internal Medicine, University of Kansas Medical Center, Kansas City, KS 66160-3018, USA; Jared Grantham Kidney Institute, University of Kansas Medical Center, Kansas City, KS 66160-3018, USA; Department of Internal Medicine, University of Kansas Medical Center, Kansas City, KS 66160-3018, USA; Jared Grantham Kidney Institute, University of Kansas Medical Center, Kansas City, KS 66160-3018, USA; Department of Internal Medicine, University of Kansas Medical Center, Kansas City, KS 66160-3018, USA; Jared Grantham Kidney Institute, University of Kansas Medical Center, Kansas City, KS 66160-3018, USA; Department of Internal Medicine, University of Kansas Medical Center, Kansas City, KS 66160-3018, USA; Jared Grantham Kidney Institute, University of Kansas Medical Center, Kansas City, KS 66160-3018, USA; Department of Molecular and Integrative Physiology, University of Kansas Medical Center, Kansas City, KS 66160-3018, USA

**Keywords:** ADPKD, CaMK4, CaMKKβ, calmodulin, LKB1, AMPK

## Abstract

Autosomal dominant polycystic kidney disease (ADPKD) is characterized by progressive enlargement of fluid-filled cysts, causing nephron loss and a decline in renal function. Mammalian target of rapamycin (mTOR) is overactive in cyst-lining cells and contributes to abnormal cell proliferation and cyst enlargement; however, the mechanism for mTOR stimulation remains unclear. We discovered that calcium/calmodulin (CaM) dependent kinase IV (CaMK4), a multifunctional kinase, is overexpressed in the kidneys of ADPKD patients and PKD mouse models. In human ADPKD cells, CaMK4 knockdown reduced mTOR abundance and the phosphorylation of ribosomal protein S6 kinase (S6K), a downstream target of mTOR. Pharmacologic inhibition of CaMK4 with KN-93 reduced phosphorylated S6K and S6 levels and inhibited cell proliferation and *in vitro* cyst formation of ADPKD cells. Moreover, inhibition of calcium/CaM-dependent protein kinase kinase-β and CaM, two key upstream regulators of CaMK4, also decreased mTOR signaling. The effects of KN-93 were independent of the liver kinase B1–adenosine monophosphate-activated protein kinase (AMPK) pathway, and the combination of KN-93 and metformin, an AMPK activator, had additive inhibitory effects on mTOR signaling and *in vitro* cyst growth. Our data suggest that increased CaMK4 expression and activity contribute to mTOR signaling and the proliferation of cystic cells of ADPKD kidneys.

## Introduction

Polycystic kidney disease (PKD) is the most common inherited renal disorder, affecting >12.5 million people worldwide and accounting for ∼9% of patients on renal replacement therapy (Martinez et al., 2013). Autosomal dominant PKD (ADPKD) is the most common form of the disease, affecting ∼1:800 people, and is caused by mutations in *PKD1* and *PKD2*, which encode polycystin-1 (PC-1) and PC-2, respectively (Grantham, 2003; Harris and Torres, 2014). ADPKD is characterized by the formation of numerous fluid-filled cysts within the kidneys due to aberrant epithelial cell proliferation and fluid accumulation driven by transepithelial fluid secretion (Grantham et al., 1995; Wallace, 2011; Harris and Torres, 2014). Autosomal recessive PKD (ARPKD) is less common (∼1:20000 live births) and is caused by mutations in *PKHD1*, which encodes fibrocystin.

Mammalian target of rapamycin (mTOR) is a serine/threonine kinase that forms the catalytic subunit of two protein complexes, known as mTOR complex 1 (mTORC1) and mTORC2 (Kim et al., 2002). mTORC1 plays a central role in the integration of nutritional signals and regulates protein translation and homeostasis, cell growth, and cell cycle progression (Torres et al., 2010; Saxton and Sabatini, 2017). Upon activation, mTOR phosphorylates ribosomal protein S6 kinase (S6K), which in turn phosphorylates S6, leading to activation of protein translation and cell proliferation. mTOR is known to be directly activated by the guanosine triphosphate (GTP)-bound form of Rheb, a small GTPase of the Ras superfamily, which is inactivated by the GTPase-activating protein (GAP) tuberin (TSC2). AKT and extracellular signal-regulated kinase (ERK) inhibit TSC2, resulting in mTORC1 activation, whereas adenosine monophosphate-activated protein kinase (AMPK) enhances GAP activity of TSC2, leading to inhibition of mTORC1.

Recent studies have highlighted the importance of mTOR activation in cell proliferation and cyst growth in PKD. Levels of mTOR, phosphorylated S6K (P-S6K), and phosphorylated S6 (P-S6) are aberrantly upregulated in renal cysts in ADPKD and PKD mouse models (Tao et al., 2005; Shillingford et al., 2006; Wahl et al., 2006), and mTOR inhibition with rapalogs reduced cyst growth and kidney enlargement and preserved renal function in PKD rodent models (Torres et al., 2010). However, the clinical value of mTORC1 inhibition using rapalogs is unclear due to intolerable side effects (Serra et al., 2010; Walz et al., 2010). Targeting upstream regulators of mTOR may provide an important therapeutic approach to reducing mTOR signaling without the adverse side effects of rapamycin analogs. AMPK activators metformin and salsalate inhibit renal mTOR activity, cell proliferation, and cyst growth in animal models of PKD (Takiar et al., 2011; Leonhard et al., 2019; Lian et al., 2019); however, the clinical use of metformin for the treatment of ADPKD has not yet been demonstrated (Hallows et al., 2021; Perrone et al., 2021; Brosnahan et al., 2022). Thus, a better understanding of the pathways that contribute to mTOR activation in renal cystic disease may be important for the development of new therapies.

Calcium/calmodulin (CaM)-dependent kinase type IV (CaMK4) is a multifunctional kinase that phosphorylates transcription factors, leading to the regulation of gene expression, metabolism, and cell proliferation (Ichinose et al., 2011b; Naz et al., 2016; Brzozowski and Skelding, 2019). Recently, CaMK4 has been reported to regulate mTOR and contribute to abnormal cell proliferation in hepatic and prostate cancer, lupus nephritis, and autoimmune diseases (Rokhlin et al., 2010; Ichinose et al., 2011a; Koga et al., 2014b; Lin et al., 2015). Kidneys of CaMK4^–/–^ mice exhibited reduced mTOR expression, suggesting that CaMK4 regulates basal mTOR levels (Zhang et al., 2014).

In this study, we discovered that CaMK4 was overexpressed in the kidneys of human ADPKD and ARPKD patients and PKD mice. To investigate the role of CaMK4, we knocked down CaMK4 expression using shRNA and examined the effects of KN-93, a CaMK4 inhibitor, on mTOR signaling, cell proliferation, and *in vitro* cyst formation in human ADPKD cells. Our data show that CaMK4 is a novel upstream regulator of mTOR and that CaMK4 inhibition may be a therapeutic approach to slowing cyst growth in ADPKD patients.

## Results

### The expression of CaMK4 in normal, ADPKD, and ARPKD human kidneys

**Figure 1:**
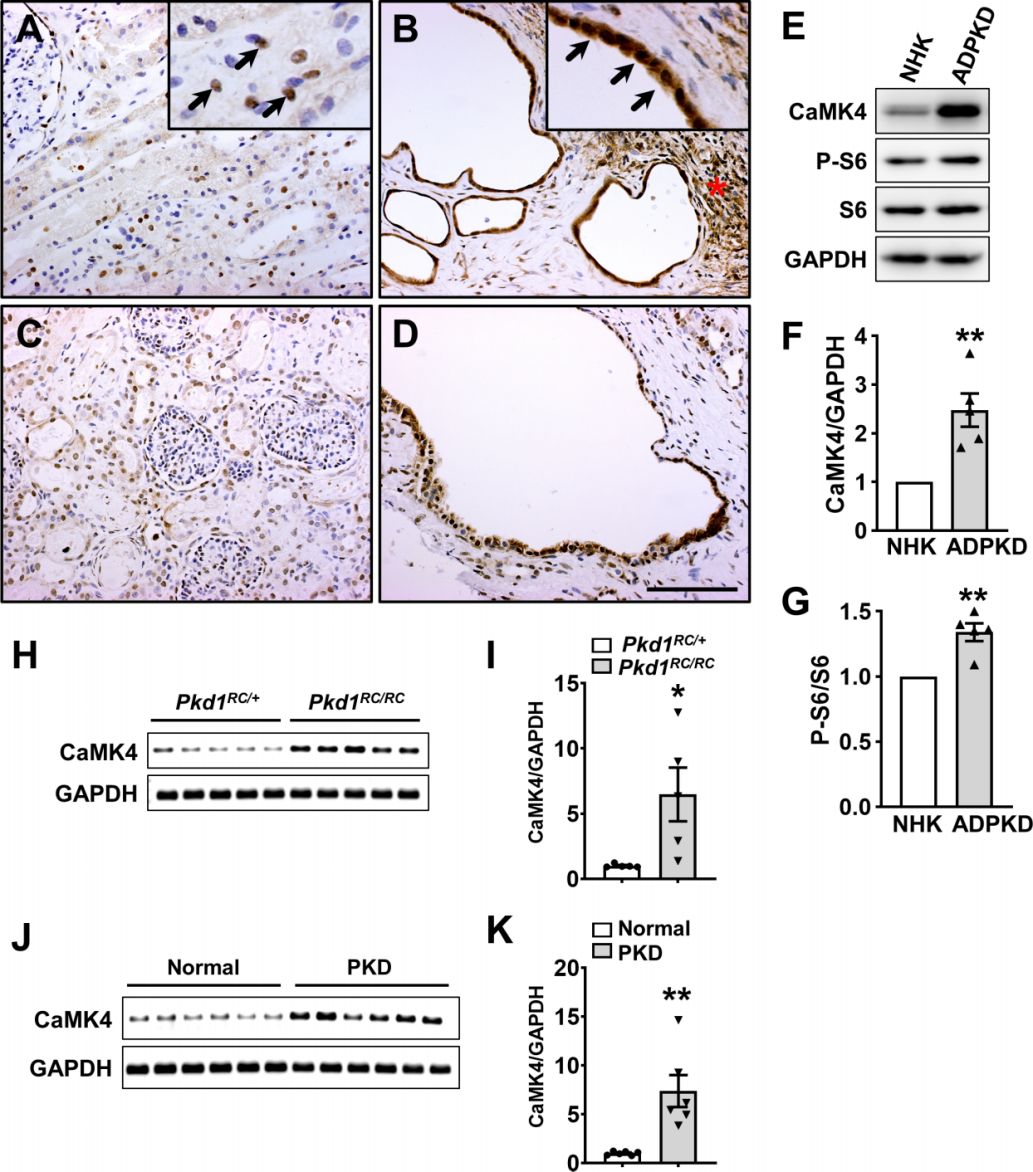
Renal expression of CaMK4 in human ADPKD and ARPKD and PKD mice. (**A**–**D**) Representative images of age- and sex-matched tissue sections stained with anti-CaMK4 antibody (brown) from an adult NHK (**A**), a human ADPKD kidney (**B**), a 4-month-old male NHK (**C**), and a 7-month-old male human ARPKD kidney (**D**). Tissues were counter-stained with hematoxylin. Images were taken at the same magnification. Scale bar, 100 μm. (**E**) Representative immunoblots for CaMK4, P-S6, S6, and GAPDH from lysates of cultured NHK and ADPKD cells. GAPDH was used as the housekeeping control. (**F** and **G**) Bars represent mean ± SEM (*n* = 5) for CaMK4/GAPDH (**F**) and P-S6/S6 (**G**). Values for ADPKD cells were normalized to NHK cells (set to 1.0) for each immunoblot. Unpaired *t*-test was used to determine statistical differences between the two groups. **P < 0.01, compared to NHK cells. (**H**) An agarose gel shows the PCR products for CaMK4 and GAPDH (40 cycles) from kidneys of 30-week-old *Pkd1^RC/RC^* mice and phenotypic normal (*Pkd1^RC/+^*) mice. (**I**) Bar graph displays mean ± SEM (*n* = 5) for the CaMK4 mRNA relative to GAPDH using qPCR. *P < 0.05, compared to normal *Pkd1^RC/+^* kidneys using an unpaired *t*-test. (**J**) An agarose gel shows the PCR products for CaMK4 and GAPDH from kidneys of *Pkd1^RC/+^:Pkd2^+/+^* (Normal) and *Pkd1^RC/RC^:Pkd2^+/–^* (PKD) mice at 20 weeks. (**K**) Bar graph shows mean ± SEM (*n* = 6) for CaMK4 mRNA relative to GAPDH. **P < 0.01, compared to normal kidneys using an unpaired *t*-test.

Normal human kidney (NHK), ADPKD kidney, and ARPKD kidney sections were stained using an antibody for CaMK4. There was moderate staining for CaMK4 in ∼30% of nuclei in renal tubules of NHK tissues with weak staining in the cytosol (Figure 1A). By contrast, age- and sex-matched ADPKD tissue sections showed intense CaMK4 staining in both the cytoplasm and nuclei of cyst-lining epithelial cells (inset, Figure 1B) and interstitial cells adjacent to cysts (asterisk, Figure 1B). Staining was not observed with a control rabbit IgG antibody (Supplementary Figure S1). There was also intense CaMK4 staining in cystic cells of a 7-month-old male human ARPKD kidney compared to an age- and sex-matched NHK (Figure 1C and D). ADPKD cells showed a 2.5-fold higher level of CaMK4 expression compared with NHK cells using immunoblot analysis (Figure 1E and F). Consistent with previous studies (Shillingford et al., 2006; Raman et al., 2017), P-S6/S6 levels were increased in ADPKD cells compared to NHK cells (Figure 1E and G). CaMK2, another member of the CaMK family, was not different between ADPKD and NHK cells (Supplementary Figure S2).

### The expression of CaMK4 in the kidneys of PKD mice

Consistent with a previous study (Arroyo et al., 2021), 30-week-old *Pkd1^RC/RC^* (*BALB/c* background) mice had significant renal cystic disease and an elevated kidney weight/body weight ratio (KW/BW; 3.26% ± 0.24% for *Pkd1^RC/RC^* mice compared with 1.33% ± 0.07% for phenotypic normal *Pkd1^RC/+^* mice, *n* = 5). We quantified renal CaMK4 mRNA levels and found a 6.4-fold increase in CaMK4/glyceraldehyde 3-phosphate dehydrogenase (GAPDH) of *Pkd1^RC/RC^* mice compared with *Pkd1^RC/+^* littermates (Figure 1H and I). *Pkd1^RC/RC^:Pkd2^+/^^–^* mice have a more rapid onset and severe disease progression of PKD (Radadiya et al., 2021). We found a 7.4-fold elevation of renal CaMK4 mRNA level in 20-week-old *Pkd1^RC/RC^:Pkd2^+/^^–^* mice compared with the phenotypic normal *Pkd1^RC/+^:Pkd2^+/+^* mice (Figure 1J and K).

### Effects of CaMK4 gene knockdown on mTOR, GSKβ, and AKT in ADPKD cells

**Figure 2:**
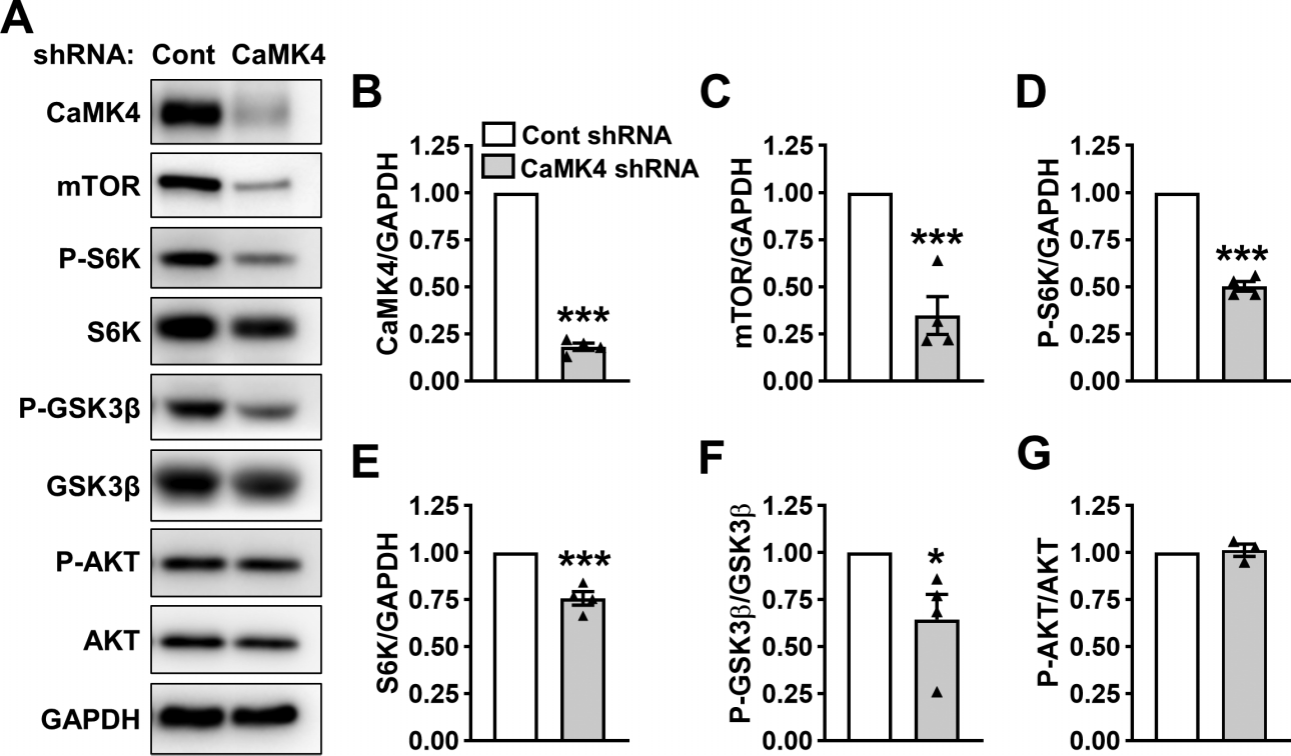
Effects of CaMK4 knockdown on mTOR abundance and downstream signaling and on phosphorylation of GSK3β and AKT in human ADPKD cells. (**A**) Representative immunoblots for ADPKD cells infected with lentivirus carrying a scrambled (Cont) or validated CaMK4 shRNA. (**B**–**G**) Bars represent mean ± SEM (*n* = 4) for CaMK4/GAPDH (**B**), mTOR/GAPDH (**C**), P-S6K/GAPDH (**D**), S6K/GAPDH (**E**), P-GSK3β/GSK3β (**F**), and P-AKT/AKT (**G**) in cells expressing the Cont or CaMK4 shRNA. Values were normalized to the group receiving the Cont shRNA (set to 1.0). Unpaired *t*-test was used to determine statistical differences between the Cont and CaMK4 shRNA groups; ***P < 0.001, *P < 0.05, compared to Cont shRNA.

Three CaMK4 shRNA lentiviral constructs were tested (Supplementary Figure S3), and one construct (#2) achieved an 80% reduction in CaMK4 expression level in ADPKD cells compared to a scrambled (control) shRNA (Figure 2A and B). CaMK4 knockdown in ADPKD cells using CaMK4 shRNA (#2) decreased mTOR abundance by 65%, P-S6K by 50%, and total S6K by 25% compared to the cells treated with control shRNA (Figure 2A and C–E). We also found that CaMK4 knockdown reduced P-GSK3β/GSK3β by 35% (Figure 2A and F) but did not affect P-AKT/AKT (Figure 2A and G).

### Effects of CaMK4 pharmacologic inhibition on mTOR signaling in ADPKD cells

**Figure 3:**
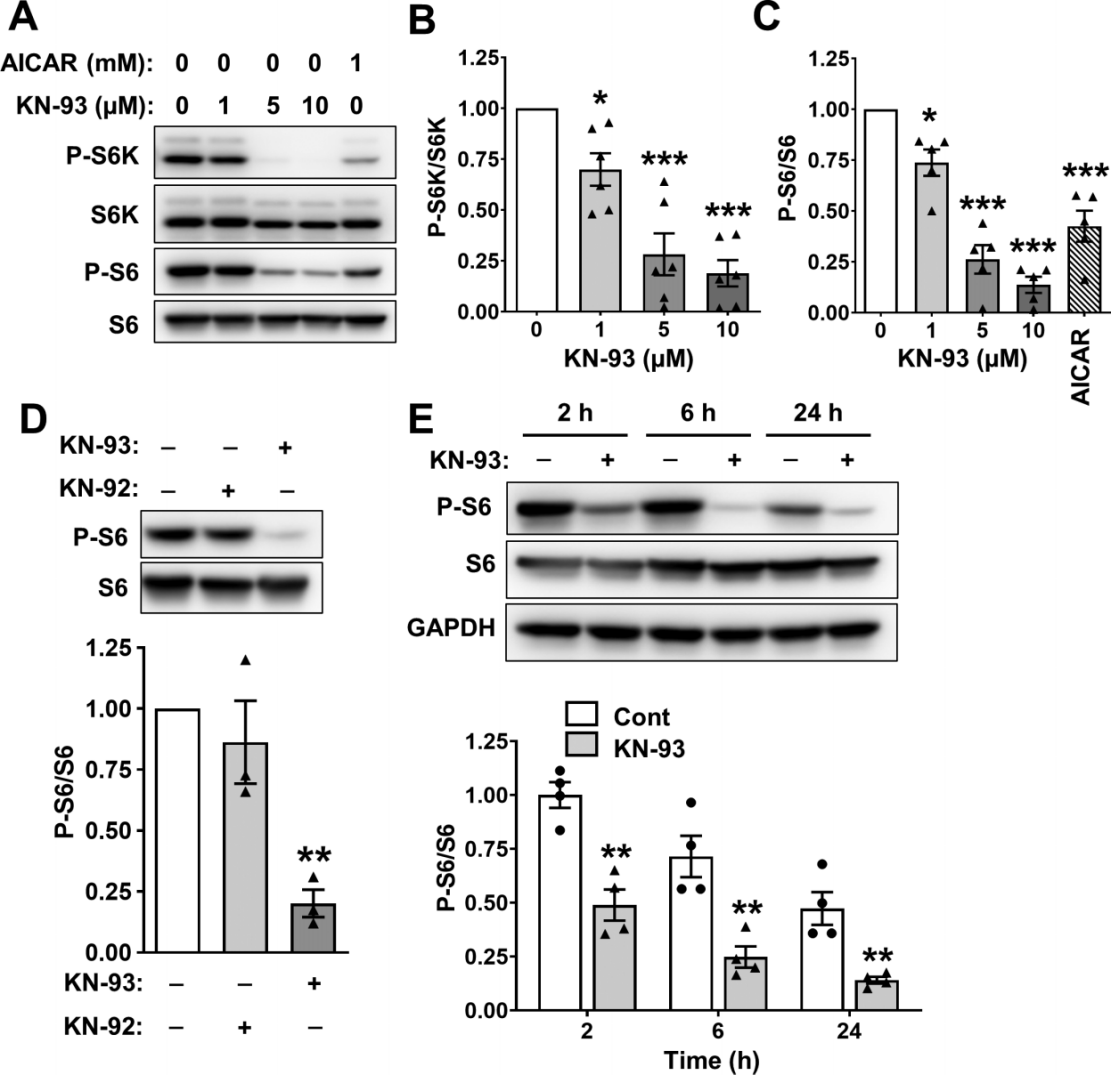
Effects of CaMK4 pharmacologic inhibition on mTOR signaling in human ADPKD cells. (**A**) Representative immunoblots for ADPKD cells treated with 0.1% DMSO (vehicle), KN-93 at concentrations ranging from 1 to 10 μM, or 1 mM AICAR, a potent AMPK activator, for 24 h. (**B** and **C**) Bar graphs display mean ± SEM for P-S6K/S6K (**B**, *n* = 6) and P-S6/S6 (**C**, *n* = 5). Values were normalized to the vehicle-treated group (set to 1.0). One-way ANOVA followed by a Student–Newman–Keuls (S–N–K) post-test was used to determine significant differences among the groups. *P < 0.05 and ***P < 0.001, compared to the vehicle group. (**D**) Representative immunoblots for ADPKD cells treated with vehicle, 10 μM KN-93, or 10 μM KN-92, an inactive analog of KN-93, for 24 h. Bar graph (lower panel) displays mean ± SEM (*n* = 3) for P-S6/S6. Values were normalized to the vehicle group (set to 1.0). One-way ANOVA followed by an S–N–K post-test was used to determine significant differences among the groups. **P < 0.01, compared to vehicle- and KN-92-treated groups. (**E**) Representative immunoblots for ADPKD cells treated with vehicle or 10 μM KN-93 for 2, 6, or 24 h. Bar graph shows mean ± SEM (*n* = 4) for P-S6/S6. Values were normalized to the Cont group at 2 h (set to 1.0). Two-way ANOVA followed by a Bonferroni post-test was used to determine significant differences between Cont and KN-93-treated groups at each time point. **P < 0.01, compared to the Cont group at the specific time point.

To determine whether pharmacologic inhibition of CaMK4 reduces mTOR-mediated proliferation of ADPKD cells, we tested KN-93, a cell-permeable inhibitor that antagonizes the binding of Ca^2+^/CaM to CaMK4 (Koga et al., 2014a; Pellicena and Schulman, 2014; Maeda et al., 2018). KN-93 treatment for 24 h decreased P-S6K/S6K and P-S6/S6 in a dose-dependent manner in ADPKD cells (Figure 3A–C), with 10 μM KN-93, the highest concentration tested, reducing P-S6K/S6K and P-S6/S6 levels by 81% and 86%, respectively. This concentration of KN-93 was more effective than 1 mM AICAR, a potent AMPK activator (Figure 3A and C). We also found that KN-93 significantly inhibited the proliferation of human ADPKD cells (Supplementary Figure S4).

Previously, KN-93 and its analog KN-92, which does not affect CaMK4 activity, were both shown to inhibit ion currents in cells overexpressing K^+^ and Ca^2+^ channels (Gao et al., 2006; Rezazadeh et al., 2006). To determine whether the effect of KN-93 was mediated by inhibition of ion channels, we compared the effects of KN-93 and KN-92 on mTOR activity in human ADPKD cells. Treatment with KN-93 for 24 h significantly reduced P-S6/S6, whereas there was no effect of KN-92 (Figure 3D). KN-93 caused a 50% reduction in P-S6/S6 within 2 h and an ∼80% reduction by 24 h (Figure 3E). We also found that treatment with 10 μM KN-93 for 6 h did not affect mTOR abundance (mTOR/ponceau S staining was 1.05 ± 0.18 compared to the vehicle-treated group set to 1.0, *n* = 3, data not shown) and P-GSK3β/GSK3β (1.0 ± 0.04 compared to the vehicle-treated group set to 1.0, *n* = 3, data not shown).

### The role of the LKB1–AMPK pathway in mTOR inhibition by KN-93 in ADPKD cells

**Figure 4:**
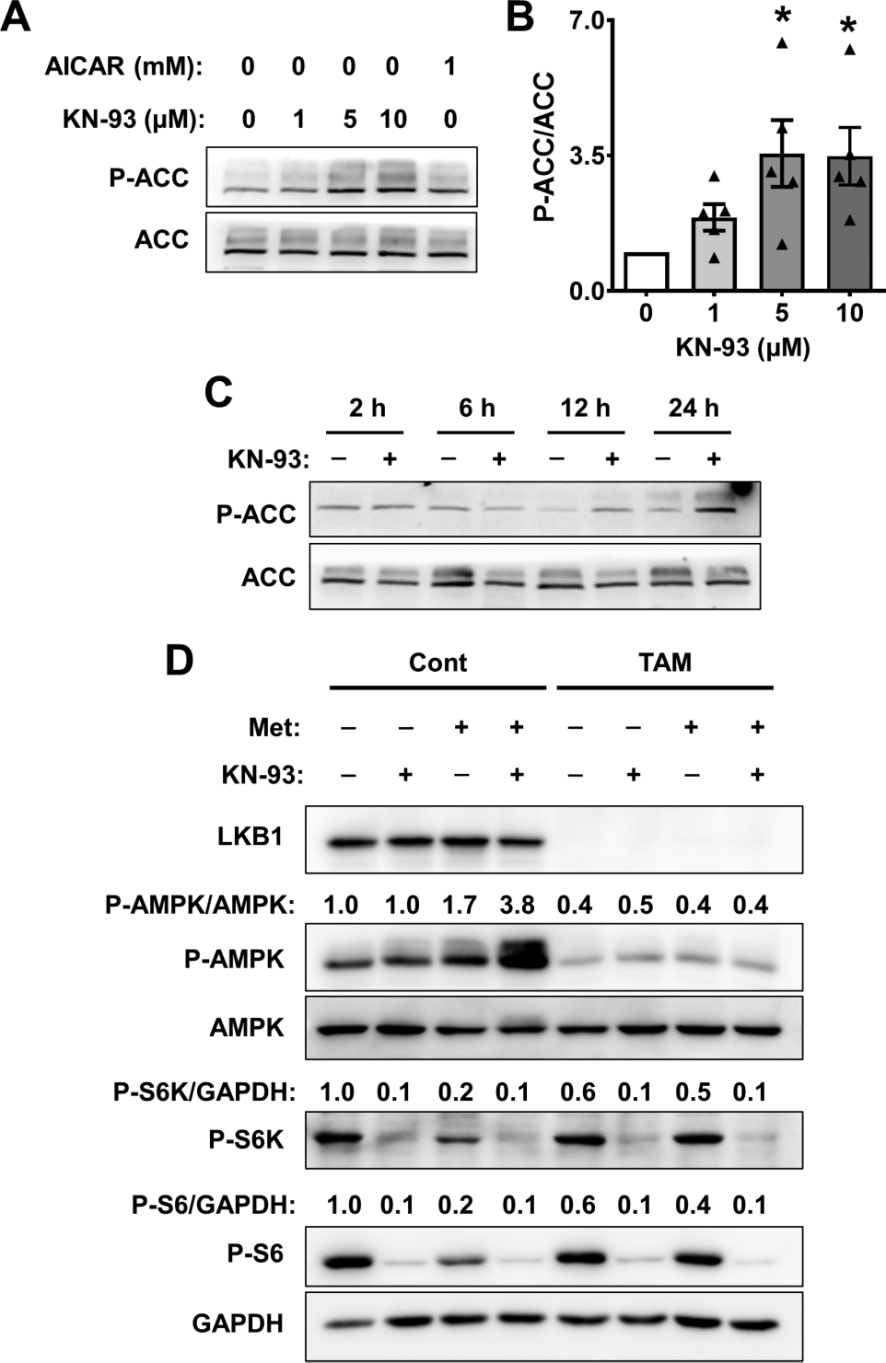
Effects of KN-93 on P-ACC levels in human ADPKD cells. (**A**) Representative immunoblots for ADPKD cells treated with 0.1% DMSO (vehicle), KN-93 at concentrations ranging from 1 to 10 μM, or 1 mM AICAR for 24 h. (**B**) Bars represent mean ± SEM (*n* = 5) for P-ACC/ACC. Values were normalized to the vehicle-treated group (set to 1.0). One-way ANOVA followed by an S–N–K post-test was used to determine significant differences among the groups. *P < 0.05, compared to the vehicle-treated group. (**C**) Representative immunoblots for P-ACC and ACC in lysates from ADPKD cells treated with vehicle or 10 μM KN-93 for 2, 6, 12, and 24 h. (**D**) Primary renal epithelial cells were isolated from 3-week-old S*kt11^fl/fl^:ROSA26-Cre^ERT2^* (conditional LKB1 knockout) mice and treated with 0.01% ethanol (vehicle control; Cont) or 1 μg/ml tamoxifen (TAM) for 48 h to knock out LKB1 expression, and then treated with 0.1% DMSO (vehicle), 10 μM KN-93, 1 mM metformin, or the combination of KN-93 and metformin for 24 h. Representative immunoblots are shown for LKB1, P-AMPK, AMPK, P-S6K, P-S6, and GAPDH. Numbers above the immunoblots are the ratios after normalization to vehicle control (set to 1.0).

KN-93 treatment for 24 h caused a significant increase in the phosphorylation of acetyl-CoA carboxylase (P-ACC), an endogenous AMPK substrate, in a dose-dependent relationship (Figure 4A and B). This effect appeared to be time-dependent, since there was no effect of KN-93 on P-ACC/ACC before 12 h, whereas there was an ∼3.5-fold increase by 24 h (Figure 4C).

To evaluate the role of liver kinase B1 (LKB1)–AMPK signaling in CaMK4 regulation of mTOR, we generated inducible LKB1 (encoded by the *STK11* gene) knockout cells from *Stk11^fl/fl^:ROSA26-Cre^ERT2^* mice. Renal epithelial cells isolated from the mice were treated with vehicle (0.01% ethanol; Cont) or 1 μg/ml tamoxifen (TAM) for 48 h to activate Cre recombinase and knock out LKB1 expression. LKB1 knockout reduced P-AMPK but did not affect basal levels of P-S6K or P-S6 (Figure 4D). As expected, 1 mM metformin, an AMPK activator, increased P-AMPK and decreased P-S6K and P-S6 in vehicle-treated control cells but not in TAM-treated LKB1 knockout cells. By contrast, KN-93 reduced P-S6K and P-S6 levels in both control and LKB1 knockout cells, demonstrating that KN-93 inhibits mTOR independent of the LKB1–AMPK pathway.

### Effects of CaMKKβ inhibition on mTOR signaling in ADPKD cells

**Figure 5:**
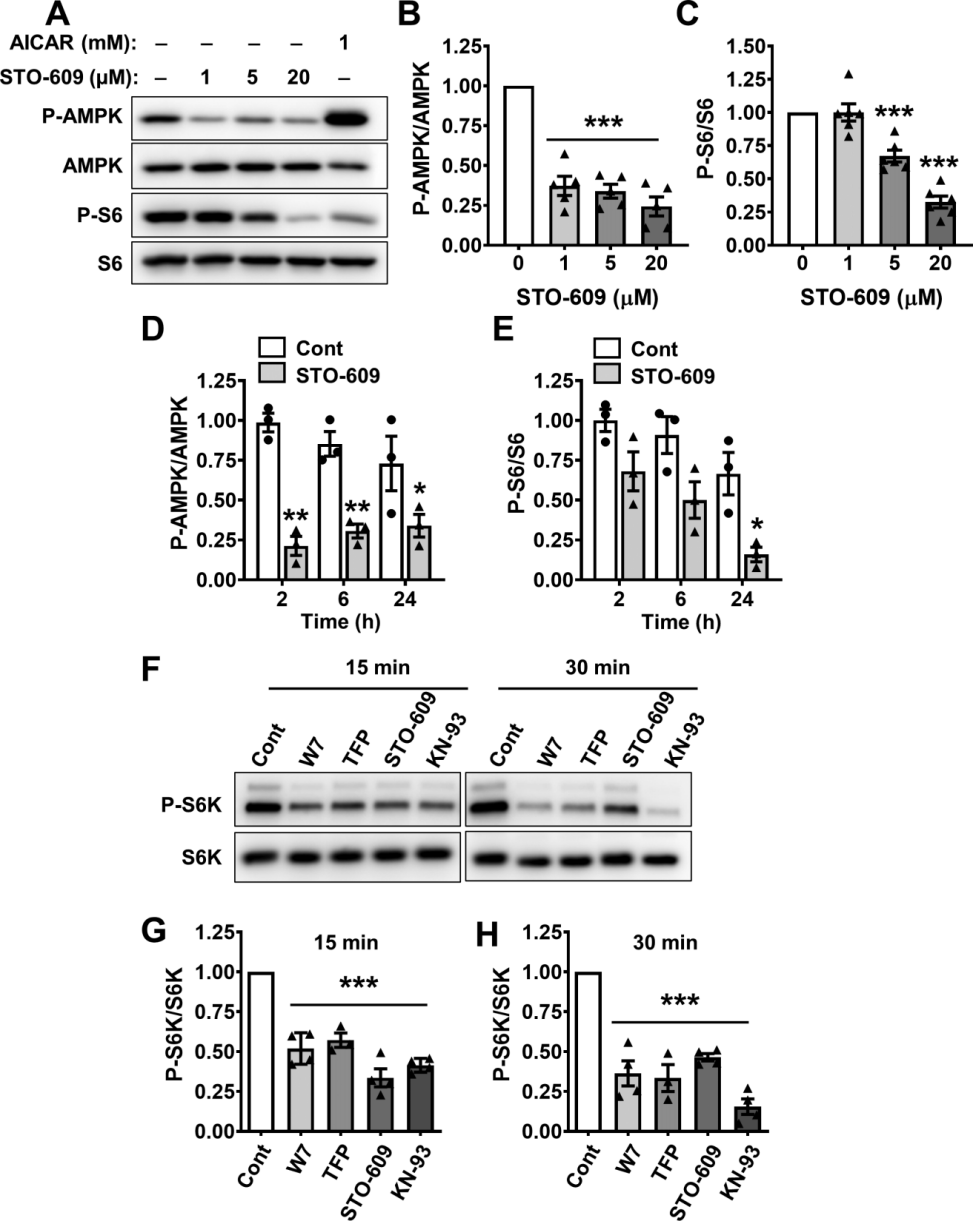
Effects of CaMKKβ inhibition on mTOR signaling in ADPKD cells. (**A**) Representative immunoblots for ADPKD cells treated with 0.1% DMSO (vehicle), the CaMKKβ inhibitor STO-609 at concentrations ranging from 1 to 20 μM, or 1 mM AICAR for 24 h. (**B** and **C**) Bars represent mean ± SEM for P-AMPK/AMPK (**B**, *n* = 5) and P-S6/S6 (**C**, *n* = 6). Values were normalized to the vehicle group (set to 1.0) for each immunoblot. Significant differences among the groups were determined by one-way ANOVA followed by an S–N–K post-test. ***P < 0.001, compared to the vehicle group. (**D** and **E**) Bar graphs show mean ± SEM (*n* = 3) for P-AMPK/AMPK (**D**) and P-S6/S6 (**E**) in ADPKD cells treated with 0.1% DMSO (Cont) or 20 μM STO-609 for 2, 6, or 24 h and determined by immunoblot analysis. Values were normalized to the Cont group at 2 h (set to 1.0) for each immunoblot. Two-way ANOVA followed by a Bonferroni post-test was used to determine significant differences between Cont and STO-609-treated groups at each time point. *P < 0.05 and **P < 0.01, compared to the Cont group at the specific time point. (**F**) Representative immunoblots for P-S6K and S6K in lysates of ADPKD cells treated with 0.1% DMSO (Cont), 30 μM W7, 10 μM TFP, 20 μM STO-609, or 10 μM KN-93 for 15 or 30 min. (**G** and **H**) Bar graphs display mean ± SEM (*n* = 4) for P-S6K at 15 (**G**) and 30 min (**H**). Values were normalized to Cont at each time point (set to 1.0). Significant differences among the groups were determined by one-way ANOVA followed by an S–N–K post-test. ***P < 0.001, compared to the Cont group, for each drug treatment group.

Calcium/CaM-dependent protein kinase kinase-β (CaMKKβ) phosphorylates and activates AMPK in certain cell types such as neuronal cells (Hawley et al., 2005) and is an important upstream regulator of CaMK4. STO-609, a cell-permeable and selective CaMKKβ inhibitor (Tokumitsu et al., 2002), decreased P-AMPK/AMPK in ADPKD cells (Figure 5A and B), as expected; however, the reduction in P-AMPK did not increase mTOR activity (Figure 5A and C). On the contrary, STO-609 decreased P-S6/S6 levels in a dose-dependent manner, similar to CaMK4 inhibition by KN-93. The effect of STO-609 on P-AMPK/AMPK was observed as early as 2 h and was sustained for 24 h (Figure 5D). STO-609 also caused a time-dependent decrease in mTOR activity with a significant reduction in P-S6/S6 at 24 h (Figure 5E). CaM directly regulates the kinase activity of CaMKKβ and CaMK4, and therefore, we evaluated the effects of CaM inhibitors W7 and trifluoperazine (TFP) on mTOR signaling. W7 and TFP significantly reduced P-S6K/S6K at 15 and 30 min, similar to the effects of KN-93 and STO-609 (Figure 5F–H). These data demonstrate that inhibition of key upstream regulators of CaMK4 also reduces mTORC1 signaling in ADPKD cells.

### Effects of AKT inhibition on CaMK4-mediated mTOR regulation in ADPKD cells

**Figure 6:**
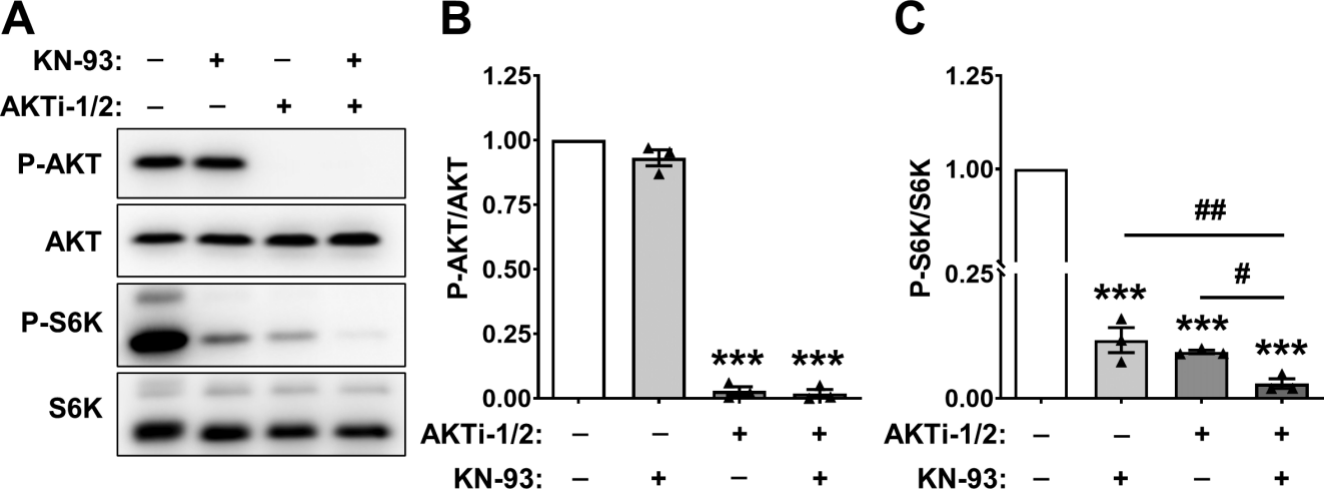
Effects of AKT inhibition on mTOR inhibition by KN-93 in ADPKD cells. (**A**) Representative immunoblots for P-AKT, AKT, P-S6K, and S6K in ADPKD cells treated with 0.01% DMSO (vehicle) or 10 μM AKTi-1/2, a potent and selective dual AKT1 and AKT2 inhibitor, for 10 min, and then treated with either 0.1% DMSO (vehicle) or 10 μM KN-93 for an additional 30 min. (**B** and **C**) Bars represent mean ± SEM (*n* = 3) for P-AKT/AKT (**B**) and P-S6K/S6K (**C**). Significant differences among the groups were determined by a one-way ANOVA followed by an S–N–K post-test. ***P < 0.001, compared to the Cont group; ^#^*P* < 0.05, AKTi-1/2-treated group vs. AKTi-1/2 plus KN-93-treated group; ^##^*P* < 0.01, KN-93-treated group vs. AKTi-1/2 plus KN-93-treated group.

CaMK4 has been reported to bind to and activate AKT, a known regulator of mTOR (Koga et al., 2014a). We found that CaMK4 knockdown did not alter P-AKT/AKT levels (Figure 2A and G). Treatment with 10 μM KN-93 for 30 min reduced P-S6K/S6K by 90% but had no effect on P-AKT/AKT (Figure 6). Treatment of ADPKD cells with an AKT inhibitor (AKTi-1/2) prior to KN-93 did not prevent the effect of KN-93 on P-S6K/S6K, but rather resulted in an additive reduction in P-S6K/S6K, indicating that CaMK4 regulation of mTOR was independent of AKT.

### Effects of CaMK4 inhibition and AMPK activation on mTOR signaling

**Figure 7:**
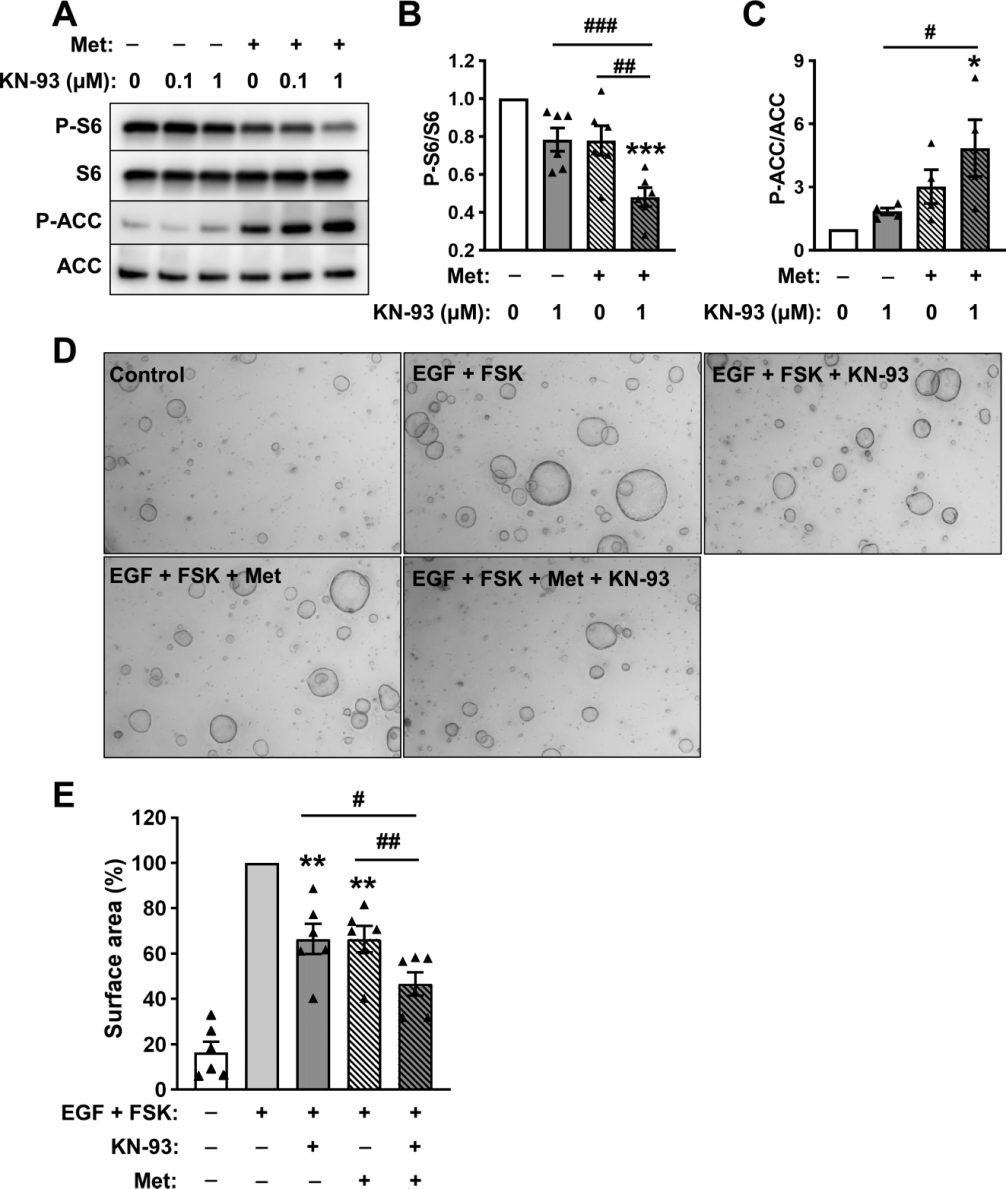
Effects of CaMK4 inhibition and AMPK activation on mTOR signaling and *in vitro* cyst growth of ADPKD cells. (**A**) Representative immunoblots of ADPKD cells were treated with 0.01% DMSO (vehicle), 0.1 or 1 μM KN-93, 0.1 mM metformin (Met), or the combination of KN-93 and Met for 24 h. (**B** and **C**) Bar graphs display mean ± SEM for P-S6/S6 (**B**, *n* = 6) and P-ACC/ACC (**C**, *n* = 4). Values were normalized to the vehicle control group (set to 1.0) for each immunoblot. One-way ANOVA followed by an S–N–K post-test was used to determine significant differences among the groups. *P < 0.05 and ***P < 0.001, compared to the vehicle control group; ^#^*P* < 0.05, KN-93 alone vs. KN-93 plus Met; ^##^*P* < 0.01, KN-93 alone vs. KN-93 plus Met; ^###^*P* < 0.001, Met alone vs. KN-93 plus Met. (**D**) Representative images of ADPKD cells cultured within collagen gels and stimulated with 5 ng/ml EGF + 5 μM FSK for 3 days to initiate *in vitro* cyst formation. After EGF and FSK were removed, the cysts were treated with control media or with 5 ng/ml EGF + 5 μM FSK alone or in combination with 1 μM KN-93, 0.1 mM Met, or KN-93 plus Met for ∼6 days. (**E**) Bars represent mean ± SEM for total cyst surface area per well of 96-well plates from six experiments using different ADPKD cell preparations. Values were normalized to EGF + FSK group (set to 100%). One-way ANOVA followed by an S–N–K post-test was used to determine significant differences among the groups. **P < 0.01, compared to the EGF + FSK group; ^#^*P* < 0.05, KN-93 alone vs. KN-93 plus Met; ^##^*P* < 0.01, Met alone vs. KN-93 plus Met.

To determine whether CaMK4 inhibition and AMPK activation can exhibit an additive inhibitory effect on mTOR signaling, we treated ADPKD cells with 1 μM KN-93, 0.1 mM metformin, or a combination of both. KN-93 and metformin individually decreased P-S6/S6 levels by ∼20%, whereas their combination resulted in a significantly greater reduction in P-S6/S6 (52.0% ± 5.0%; Figure 7A and B). Metformin and KN-93 independently increased P-ACC/ACC, and the effect was greater with their combination (Figure 7A and C). These data indicate that KN-93 and metformin have additive effects on mTOR inhibition and AMPK activation.

### Effects of CaMK4 inhibition and AMPK activation on *in vitro* cyst growth of ADPKD cells

To test the effects of CaMK4 inhibition and AMPK activation on *in vitro* cyst formation, ADPKD cells were seeded within a collagen matrix and stimulated to form cysts with the treatment of epidermal growth factor (EGF) and forskolin (FSK), an activator of adenylyl cyclase. EGF and FSK increased the size and number of cysts (Figure 7D). The average total surface area of cysts per well was normalized to the EGF and FSK group (set to 100%) for each experiment. Treatment with 1 μM KN-93 or 0.1 mM metformin individually reduced total cyst surface area per well by 34%, and the combination resulted in a greater inhibition (53.4% ± 5.2%; Figure 7E). Treatment with 20 μM STO-609 or 10 μM W7 completely abolished the enlarged total cyst surface area per well by EGF and FSK stimulation (Supplementary Figure S5).

## Discussion

CaMK4 was discovered to be overexpressed in the kidneys of human ADPKD and ARPKD patients and PKD rodent models with intense staining in the cystic epithelia. CaMK4 knockdown reduced mTOR and P-S6K levels, which were accompanied by a reduction in the phosphorylation of GSK3β, at an inhibitory residue. Pharmacologic inhibition of CaMK4 with KN-93 reduced levels of P-S6K and P-S6, and inhibited ADPKD cell proliferation and *in vitro* cyst growth. The inhibitory effects of KN-93 on mTOR signaling and cyst growth were enhanced with metformin, an AMPK activator. These data suggest that CaMK4 is an important upstream regulator of mTOR and may be a potential therapeutic target to reduce mTOR-dependent cyst growth in ADPKD. A limitation of the study is that the conclusions are based on *in vitro* experiments using cultured human ADPKD cells and rely on pharmacological inhibitors. Future studies will need to be conducted with genetically altered expression of CaMK4 in the kidneys of wild-type and PKD mice.

Intracellular Ca^2+^ is an essential signaling molecule that forms a complex with calcium-binding proteins, one of the most important being CaM (Yamniuk et al., 2007). Ca^2+^ binding to CaM induces a conformational change, increasing its affinity for downstream targets. The Ca^2+^/CaM complex binds to and activates members of the CaMK family, including CaMKKα, CaMKKβ, CaMK1, CaMK2, and CaMK4. Full CaMK4 activation requires Ca^2+^/CaM binding and phosphorylation by CaMKKβ (Anderson and Kane, 1998). Upon activation, CaMK4 translocates to the nucleus, where it phosphorylates and regulates transcription factors, including cyclic AMP-response element-binding protein, AP-1, myocyte enhancer factor 2A, and retinoid orphan receptor family members (Racioppi and Means, 2008). CaMK4 is involved in transcriptional regulation in lymphocytes, neurons, and male germ cells (Naz et al., 2016), and its overexpression has been implicated in a variety of human diseases characterized by high-level cell proliferation and/or inflammation. Suppression of CaMK4 can mitigate these diseases and, hence, is considered a potential therapeutic target (Maeda et al., 2018; Brzozowski and Skelding, 2019).

Recently, CaMK4 has been shown to regulate mTOR abundance and signaling in multiple cell types, and the mechanism seems to be cell-specific. In myeloid leukemia cells and macrophages, CaMK4 phosphorylates and inhibits GSK3β, preventing proteasomal degradation of mTOR (Si and Collins, 2008; Zhang et al., 2014). CaMK4 was also shown to increase mTOR signaling through the phosphorylation and activation of AKT in IL-17-producing T helper (Th17) cells during cellular differentiation (Koga et al., 2014a). In hepatic cancer cells, CaMK4 and CaMKKβ provide a scaffold for the recruitment and coordinated regulation of the mTOR–S6K complex to control protein synthesis (Lin et al., 2015).

Elevated mTOR activation is an established feature of PKD and is thought to contribute importantly to cell proliferation and cyst growth (Shillingford et al., 2006; Wu et al., 2007; Torres et al., 2010; Ravichandran et al., 2014); however, the mechanism responsible for elevated mTOR activity remains unclear. TSC2 directly interacts with PC-1 to inhibit mTOR signaling, and the loss of PC-1 in ADPKD is thought to be attributed to inappropriate mTOR activity (Brook-Carter et al., 1994; Shillingford et al., 2006). Previously, we showed that intracellular cyclic AMP activates BRAF, an upstream kinase of MEK, causing elevated ERK signaling (Yamaguchi et al., 2004, 2010). ERK directly phosphorylates and inactivates TSC2, increasing mTOR signaling (Ma et al., 2005). Evidence has also shown that PC-1 controls mTOR signaling in a TSC2-dependent manner by inhibiting ERK phosphorylation of TSC2 (Distefano et al., 2009). Other studies have suggested that reduced LKB1–AMPK signaling due to changes in cellular metabolism, including aerobic glycolysis, or lower LKB1 activity due to phosphorylation of an inhibitory site contributes to elevated mTOR signaling (Rowe et al., 2013; Lian et al., 2017).

To our knowledge, this is the first study to demonstrate that CaMK4 is overexpressed in PKD and contributes to increased mTOR signaling and proliferation of cystic epithelial cells. Elevated CaMK4 expression in cyst-lining cells of human ADPKD, ARPKD, and rodent models of PKD indicates that CaMK4 overexpression is a common feature of renal cystic disease regardless of the gene mutation. CaMK4 expression was also elevated in the interstitium adjacent to cysts, possibly due to the activation of inflammatory cells in the cystic kidneys (Swenson-Fields et al., 2013; Kleczko et al., 2018; Li et al., 2021). While this study did not specifically investigate the role of CaMK4 in inflammation, the kinase has been reported to regulate macrophage autophagy, Th17 cell differentiation, and myofibroblast activation (Ao et al., 2013; Koga et al., 2014a; Zhang et al., 2014). Further studies are required to determine the impact of CaMK4 overexpression on renal inflammation and fibrosis in PKD.

Using genetic and pharmacological approaches, we showed that CaMK4 is an important regulator of mTOR-mediated proliferation of human ADPKD cells and that inhibition of CaMK4 or its upstream regulators CaM and CaMKKβ significantly reduced mTOR signaling. KN-93 is the most commonly used pharmacologic inhibitor for the investigation of CaMK4 (Koga et al., 2014a, b; Ferretti et al., 2018; Maeda et al., 2018); however, the compound can also inhibit CaMK2. Therefore, we cannot exclude the possibility that changes in CaMK2 activity contribute to the inhibitory effect of KN-93 on mTOR signaling or cell proliferation. However, unlike CaMK4, CaMK2 expression is not elevated in PKD kidneys. KN-93 has also been reported to inhibit membrane currents of cells overexpressing K^+^ and Ca^2+^ channels through a mechanism that is independent of CaMK4 or CaMK2 (Gao et al., 2006; Rezazadeh et al., 2006). We found that KN-92, an analog that lacks kinase activity but retains the capacity to inhibit K^+^ or Ca^2+^ channels, did not inhibit mTOR signaling in ADPKD cells.

**Figure 8:**
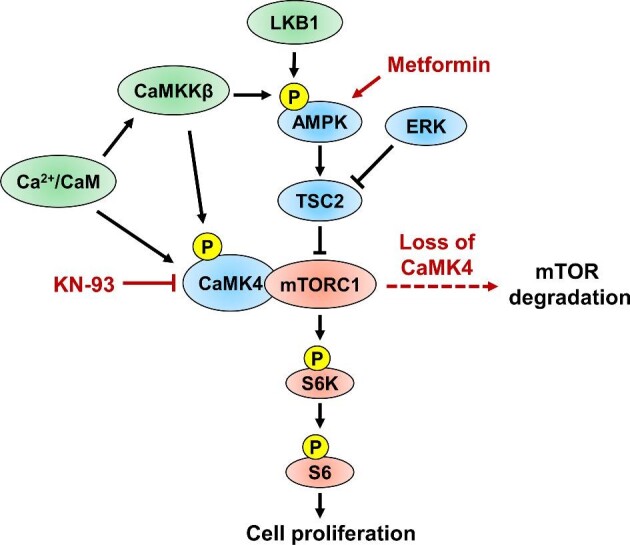
Proposed model for CaMK4 regulation of mTOR-mediated cell proliferation in ADPKD. Cyst epithelial cells have abnormally elevated expression of CaMK4, which is activated by Ca^2+^/CaM binding and the phosphorylation by CaMKKβ, a kinase that also regulates AMPK. CaMK4 stabilizes mTORC1 and contributes to the regulation of downstream targets-involved mTOR-mediated cell proliferation. CaMK4 effects on mTOR signaling are independent of LKB1 and AMPK. CaMK4 inhibition with KN-93 decreases mTOR signaling and cell proliferation, and the loss of CaMK4 expression destabilizes mTORC1, leading to mTOR degradation, possibly through GSK3β signaling and the proteasome. Metformin, a clinically relevant AMPK activator, increases AMPK phosphorylation by LKB1 and CaMKKβ, leading to decreased mTOR signaling and decreased cell proliferation. The beneficial effects of metformin are enhanced by CaMK4 inhibition with KN-93.

CaMK4 regulation of mTOR in ADPKD cells appears to be independent of AKT and the LKB1–AMPK pathway. Interestingly, CaMK4 knockdown reduced mTOR abundance and caused a small but significant decrease in P-GSK3β (Ser9), whereas CaMK4 inhibition with KN-93 inhibited mTOR signaling without affecting mTOR abundance or the level of P-GSK3β. We propose that CaMK4 interacts with and stabilizes mTORC1 and regulates mTOR signaling (Figure 8). Loss of CaMK4 destabilizes the complex, and mTOR is degraded by a mechanism regulated by GSK3β (Si and Collins, 2008; Zhang et al., 2014). Additional studies are needed to delineate the interactions between CaMK4, GSK3β, and mTOR in the setting of renal cystic disease.

CaMKKβ is another known kinase involved in AMPK activation (Woods et al., 2005; Yamauchi et al., 2008; Ma et al., 2012). In a previous study, pharmacologic activation of CaMKKβ increased P-AMPK and inhibited *in vitro* cyst formation of Madin–Darby canine kidney cells (Pathomthongtaweechai et al., 2020). We found that CaMKKβ inhibition using STO-609 strongly reduced P-AMPK levels in human ADPKD cells, suggesting that CaMKKβ is an important upstream regulator of basal AMPK activity in cystic cells. Unexpectedly, CaMKKβ inhibition with STO-609 did not increase mTOR activity but instead caused a strong inhibitory effect. Considering that CaMKKβ phosphorylation is required for maximal CaMK4 activation, we think that the effects of STO-609 on mTOR signaling are mediated through CaMK4 inhibition.

AMPK activators, such as metformin, AICAR, and salsalate, inhibit pathways involved in ADPKD progression, including mTOR-mediated cell proliferation, CFTR-mediated fluid secretion, inflammation, and fibrosis (Hallows et al., 2003; Takiar et al., 2011; Leonhard et al., 2019; Lian et al., 2019; Song et al., 2020). Our data indicate that CaMK4 inhibition of mTOR is independent of the LKB1–AMPK pathway and that AMPK activation occurs later as a secondary effect of mTOR inhibition (Rowe et al., 2013).

The therapeutic value of metformin for the clinical treatment of ADPKD is currently being tested (Hallows et al., 2021; Perrone et al., 2021; Brosnahan et al., 2022). While metformin is generally well tolerated, there are concerns that lactic acidosis in renal compromised patients may prevent the use of an optimal dose to achieve therapeutic effects (Perrone et al., 2021). Here, we showed that inhibition of CaMK4 and its upstream regulators, CaMKKβ and CaM, individually inhibited mTOR activity and *in vitro* cyst growth of the ADPKD cells. The effects of metformin on AMPK activation, mTOR inhibition, and *in vitro* cyst formation were significantly enhanced by CaMK4 inhibition with KN-93. It is possible that effective therapeutic inhibition of mTOR and activation of AMPK can be achieved with a lower dose of metformin if delivered with a small-molecule inhibitor of CaMK4 or CaMKKβ.

## Materials and methods

Full methods are available in Supplementary material. CaMK4 expression in tissue sections of NHK, ADPKD, and ARPKD kidneys was examined using immunohistochemistry. The levels of CaMK4 in NHK and ADPKD primary cells were also measured by immunoblot analysis. CaMK4 mRNA levels in *Pkd1^RC/RC^, Pkd1^RC/RC^:Pkd2^+/^^–^*, and normal mouse kidneys were quantified by quantitative polymerase chain reaction (qPCR).

Primary cells were generated from human ADPKD kidneys as previously described (Raman et al., 2018; Wallace and Reif, 2019). The effects of CaMK4 knockdown and pharmacologic inhibition of CaMK4 by KN-93 on the levels of mTOR, P-S6K, and P-S6 were examined in ADPKD cells. Effects of CaM inhibitors W7 and TFP and the CaMKKβ inhibitor STO-609 on P-S6K levels were also investigated in ADPKD cells. W7, TRP, and STO-609 were purchased from Tocris Bioscience.

To generate LKB1 knockout renal cells, *Stk11^fl/fl^* and *ROSA26-Cre^ERT2^* mice (The Jackson Laboratory) were bred to generate *Stk11^fl/fl^:ROSA26-Cre^ERT2^* mice, and renal epithelial cells were isolated from 3-week-old mice and grown in culture. These cells were treated with TAM to knock out LKB1 expression. To determine whether CaMK4 regulates mTOR via the LKB1–AMPK pathway, the effects of KN-93 on P-S6K and P-S6 were examined in LKB1 knockout cells. The AKT inhibitor AKTi-1/2 (Tocris Bioscience) was used to determine whether CaMK4 regulates mTOR through AKT.

Effects of CaMK4 inhibition on proliferation and *in vitro* cyst formation of ADPKD cells were tested. Whether the combination of CaMK4 inhibition and AMPK activation can exhibit additive effects on mTOR inhibition, AMPK activation, and *in vitro* cyst formation was also investigated.

## Supplementary Material

mjac050_Supplemental_File
