# Supplementary material for: CaMK4 overexpression in polycystic kidney disease promotes mTOR-mediated cell proliferation
Source: J Mol Cell Biol. 2022 Aug 24;14(7):mjac050. doi: 10.1093/jmcb/mjac050 (PMC9802383; doi:10.1093/jmcb/mjac050)
Supplement: mjac050_Supplemental_File [file mjac050_supplemental_file.pdf]

Supplementary material

CaMK4 overexpression in polycystic kidney disease promotes mTOR-mediated cell proliferation

Yan Zhang[1,3], Emily A. Daniel[1,3], July Metcalf[1,3], Yuqiao Dai[1,3], Gail A. Reif[1,3], and Darren P. Wallace[1,2,3,*]

[1] Department of Internal Medicine, University of Kansas Medical Center, Kansas City, KS 66160-3018, USA

[2] Department of Molecular and Integrative Physiology, University of Kansas Medical Center, Kansas City, KS 66160-3018, USA

[3] Jared Grantham Kidney Institute, University of Kansas Medical Center, Kansas City, KS 66160-3018, USA

* Correspondence to: Darren P. Wallace, Jared Grantham Kidney Institute, Department of Internal Medicine, University of Kansas Medical Center, 3901 Rainbow Boulevard, Kansas City, KS 66160-3018, USA; E-mail: dwallace@kumc.edu

Table of contents

Supplementary Methods

Supplementary Figures

Supplemental Figure S1. Validation of anti-CaMK4 antibody for immunohistochemistry.

Supplemental Figure S2. Expression of CaMK2 in primary normal human kidney (NHK) and ADPKD cells.

Supplemental Figure S3. CaMK4 knockout using shRNA in primary ADPKD cells**.**

Supplemental Figure S4. Effect of KN-93 on cell proliferation of ADPKD cells.

Supplemental Figure S5. Effects of W7, STO-609, and metformin on *in vitro* cyst formation of human ADPKD cells.

Supplementary References

Supplementary Methods

Animal care and protocol

Pkd1$^{RC/RC}$ mice (provided by Dr. Peter Harris) were backcrossed to *BALB/cByJ* mice (The Jackson Laboratory) for 10 generations. *Pkd1*$^{RC/+}$ littermates were used as phenotypic wildtype (WT) controls. *Pkd1*$^{RC/RC}$ mice were also crossed with *Pkd2*$^{+/-}$ (provided by Dr. Stefan Somlo) to generate a PKD mouse model with a rapid and onset and severe disease progression (Gainullin et al., 2015; Radadiya et al., 2021). *Pkd1*$^{RC/+}$: *Pkd2*$^{+/+}$ were used as phenotypic WT controls. *Pkd1*$^{RC/RC}$ mice and *Pkd1*$^{RC/+}$ littermates were euthanized at 30 weeks. *Pkd1*$^{RC/+}$: *Pkd2*$^{+/+}$ and *Pkd1*$^{RC/RC}$: *Pkd2*$^{+/-}$ mice were euthanized at 20 weeks.

To generate *Skt11*$^{fl/fl}$:*ROSA26-Cre*^{ERT2} mice, *Skt11*$^{fl/+}$ mice were purchased from The Jackson Laboratory (Farmington, CT) and bred with *ROSA26-Cre*^{ERT2} mice (The Jackson Laboratory). *Lkb1*$^{fl/fl}$: *ROSA26-Cre*^{ERT2} mice were euthanized at 3 weeks and kidneys were removed for isolation of primary epithelial cells as previously described (Pinto et al., 2016). The Institutional Animal Care and Use Committee of the University of Kansas Medical Center approved the protocols for the use of these mice (Protocol no. 2021-2605).

Genotyping of mice

We used a PCR method for genotyping mice as previously described (Zhang et al., 2020). Tail DNA was extracted using Extraction Solution (E7526, Sigma-Aldrich, St. Louis, MO) and Tissue Preparation Solution (T3073, Sigma-Aldrich), and then neutralized by Neutralization Solution B (N3910, Sigma-Aldrich). REDExtract-N-Amp™ PCR ReadyMix™ (R4775, Sigma-Aldrich) was used to perform PCR. For genotyping *Pkd1*$^{RC/RC}$, the forward primer (FP) sequence was 5′-GGTTCCTTAATCCTGTTGGTGTACAGCTGC-3′, and the reverse primer (RP) was 5′-CTATTTGGCTGTCCTCTTCCG-3′. For genotyping *Pkd2* mutation, the FP was 5′-GCGCCGGCCTAGCTGTCCC-3′ and RF was 5′-GTTGTCGCGGCTCCACG-3′. For genotyping *ROSA26-Cre*^{ERT2}, the FP was 5′-GCGGTCTGGCAGTAAAAACTATC-3′ and RF was 5′-GTGAAACAGCATTGCTGTCACTT-3′.

Human ADPKD and NHK cells

Primary cultures of ADPKD and NHK cells were generated by PKD Biomarkers and Biomaterials Core in the Kansas PKD center at the Kansas University Medical Center (KUMC). ADPKD kidneys were obtained from the Surgery Department at KUMC with the assistance of the KU Cancer Center's Biospecimen Resource Core, and from hospitals participating in the Tissue Donation Program at the PKD Foundation (Kansas City, MO). Average age of patients at the time of nephrectomy was ~53 year

(29–73 year), and most patients were at or near end-stage renal disease (ESRD). Most, if not all samples, were from *PKD1* patients with since 85% of cases are *PKD1* mutations, and individuals with *PKD2* mutations has a milder phenotype with later onset of ESRD (54 year for *PKD1 vs*. 74 year for *PKD2* mutations) (Pei, 2011). NHK tissues were obtained from nephrectomy specimens by the Surgery Department at KUMC or from the Midwest Transplant Network (Kansas City, KS), an organ retrieval agency. The protocol for the use of the discarded human tissues for research complies with federal regulations and was approved by the Institutional Review Board at KUMC.

Primary cell cultures were prepared as described previously (Pinto et al., 2016). Cells were propagated in DMEM/F12 supplemented with 5% FBS, 5 μg/ml insulin, 5 μg/ml transferrin, and 5 ng/ml sodium selenite (ITS) and penicillin G and streptomycin (P/S). After cells had reached 70-80% confluence in T-75 tissue culture flasks, they were lifted with a trypsin-EDTA solution (T3924, Sigma-Aldrich) and counted using the TC20 automated cell counter (BioRad, Hercules, CA). Cells were not passaged more than twice before being used in experiments.

CaMK4 knockdown in primary human ADPKD cells

CaMK4 was knocked down in human ADPKD cells using an shRNA lentiviral approach as previously described (Raman et al., 2017). Three CaMK4 shRNA constructs (RHS4330), each with high specificity for the CaMK4 mRNA, and a scrambled non-silencing shRNA construct (RHS4346) were purchased from GE Healthcare (Aurora, OH). HEK293T cells were transfected with 4.5 μg scrambled or CaMK4 shRNA plasmid and the packaging vectors (4.5 μg psPAX2 and 1.8 μg pMD2.G) using Lipofectamine 200 (Thermo Scientific, Rockford, IL) and were incubated for 18 hours for lentivirus production. Then cells were incubated with fresh media for 24 hours before the conditioned media containing the lentivirus was harvested.

Lentiviral harvest media was diluted 1:500 and added to incubation media for cultured ADPKD cells. After 24 hours, media was switched to DMEM/F12, ITS, 1% FBS, and P/S containing 100 ng/ml puromycin, the minimum concentration of puromycin to eliminate non-transduced ADPKD cells (determined by a kill curve). Media with puromycin was changed every 2 days for approximately 7 days to kill non-transduced cells. Cells with successful infection were assessed by examination of GFP expression. CaMK4 knockdown efficiency was determined by immunoblot analysis. shRNA (mature antisense sequences for CaMK4 shRNA #2: 5′-ATATCTGCACCTTTAACTT-3′), which achieved an 80% knockdown efficiency, was used for the rest of the experiments.

Immunohistochemistry

Kidney tissues (~1 cm^3) were fixed in 4% paraformaldehyde overnight and embedded in paraffin blocks, and 5 µm sections were placed on glass slides. As described previously (Raman et al., 2018), kidney sections were deparaffinized, rehydrated, blocked, and incubated with the primary antibody to CaMK4 (68218, Abcam, Cambridge, MA) overnight at 4 ℃. Rabbit IgG (10500C, Thermo Scientific, Rockford, IL) was used as a negative control to demonstrate the antigen specificity (Supplemental Fig. S1A and B). Sections were rinsed with PBS-T (0.5% Tween 20 in PBS) for three times, and then incubated with ImmPRESS™ HRP reagent Anti-Rabbit IgG (peroxidase) Polymer (MP-7401, Vector Laboratories, Burlingame, CA) for 30 min at room temperature, and the antigens were detected by ImmPACT™ DAB peroxidase substrate kit (SK-4105, Vector Laboratories). Sections were counter-stained with a hematoxylin solution.

Quantitative RT-PCR

Total RNA was isolated from mouse kidneys using RNeasy isolation kit (74104, Qiagen). First-strand cDNA was synthesized from 1 µg of total RNA using the High-Capacity cDNA reverse transcription kit (4368814, Thermo Scientific). PCR reaction contained cDNA as templates, 500 nM of forward and reverse primers, and 1× SYBR Green PCR master mix (4367659, Thermo Scientific) in 20 µl and total cycles was 40 cycles. Real-time PCR reactions were performed in StepOnePlus™ Real-Time PCR cycler (4376600, Thermo Scientific). The threshold cycle (C_t) of CaMK4 gene product was normalized to the C_t of GAPDH. Primer sequences were as follows: CaMK4, forward 5’- GCTGGGACGGGGTGC-3’ and reverse 5’-TGTTCGGGTGTGAGAGACG-3’; GAPDH, forward 5’-CCACTCACGGCAAATTCAAC-3’ and reverse 5’-GTAGACTCCACGACATACTCA-3’.

Immunoblot analysis

Mouse kidneys were removed for protein extraction and immunoblot analysis as previous described (Zhang et al., 2020). Cell lysates were prepared in Triton lysis buffer with protease inhibitors (2 mM PMSF, protease inhibitor complex [P8340, Sigma]), phosphatase inhibitor cocktail 1 and 2 (P2850, P5726, Sigma). Total protein concentration was quantified using Pierce™ Detergent Compatible Bradford Assay (Thermo Scientific). Total protein (20 µg) was loaded into each well of the gel and separated by electrophoresis and transferred to nitrocellulose blotting membrane for immunoblot analysis. The following antibodies were used CaMK4 (610275, BD Biosciences, San Jose, CA[BD]), CaMK2 (611292, BD), mTOR (2972, Cell Signaling Technology, Beverly, MA[CS]), P-S6K (Thr389) (9234, CS), S6K (9292, CS), P-S6 (Ser235/236) (2211, CS), S6 (2217, CS), P-ACC (Ser79) (11818, CS), ACC

(3676, CS), P-AMPK (Thr172) (2535, CS), AMPK (2532, CS), LKB1 (3047, CS), P-AKT (Ser473) (9271, CS), AKT (9272, CS), P-GSK3β(Ser9) (9336, CS), GSK3 β (610201, BD), HRP conjugated GAPDH (HRP-60004, Proteintech, Rosemont, IL) and HRP conjugated α-tubulin (HRP-66031, Proteintech). Blots were incubated with HRP-conjugated donkey anti-rabbit IgG (NA9340V; GE Healthcare) or HRP-conjugated goat anti-mouse IgG1 (1070-05, Southernbiotech; Birmingham, AL). Bands were detected with ECL Substrate Kit (SuperSignal™ West Pico PLUS Chemiluminescent or SuperSignal™ West Femto Maximum Sensitivity, ThermoScientific), imaged using Amersham Imager 600, and quantified by AzureSpot analysis software.

Measurement of in vitro ADPKD cysts

As previously described (Sharma et al., 2019), primary cultures of ADPKD cells (5–6 $\times 10^3$ cells/well) were dispersed within a type I collagen matrix (PureCol, Advanced BioMatrix, Inc.) in individual wells of 96-well plates. Warming the plate to 37 ℃ caused polymerization of the collagen, trapping the cells within the gel. A defined media (DMEM/F12 with ITS, 5 $\times 10^{-8}$ M hydrocortisone, and 5 $\times 10^{-5}$ M triiodothyronine) supplemented with 5 μM FSK (F6886 or 344282, Sigma) and 5 ng/ml EGF (E1257, Sigma) was added for 3 days to initiate cyst growth. To test the effects of inhibitors on cyst growth of ADPKD cells, media was replaced with define media alone, or media with 5 μM FSK and 5 ng/ml EGF ± 1 μM KN-93, or 0.1 mM metformin, or KN-93 plus metformin. Media was changed every 2 or 3 days for approximately 6 days followed by fixation with 1% formalin. To create an image of all cysts in a single plane, we used the *Live Extended Depth of Field* capture mode of Image-Pro Premier (Media cybernetics, Inc.). For each cyst with a diameter of ≥ 50 μm, the average diameter of two perpendicular diameters was used to calculate a spherical surface area. Total cyst surface area per well was measured, and the mean and SEM were calculated from five or six wells per experimental group. The experiments were repeated with six ADPKD cell preparations.

Statistics

Data are expressed as mean ± standard error of mean (SEM). Statistical significance was determined using an unpaired t-test for comparison between two groups. For multiple experimental conditions, we used a one-way ANOVA followed by Student-Newman-Keuls (S-N-K) or two-way ANOVA followed by Bonferronic post-test. $P < 0.05$ was taken to be significant.

Supplementary Figures

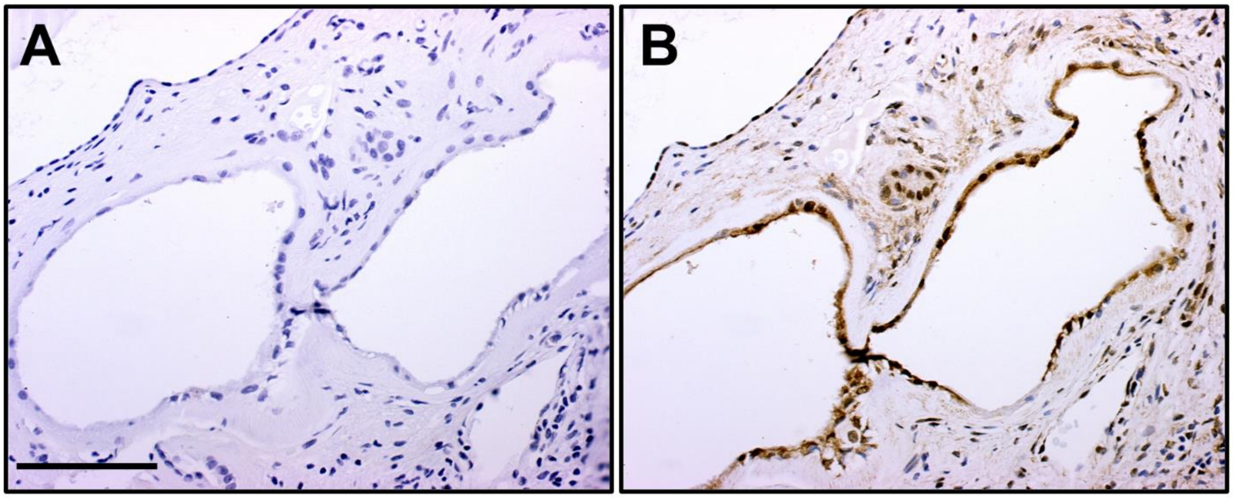

Supplemental Figure S1. Validation of anti-CaMK4 antibody for immunohistochemistry. Representative images of human ADPKD tissue sections stained with rabbit control IgG (**A**) or anti-CaMK4 antibody (**B**). Tissues were counter-stained with hematoxylin. Images were taken at the same magnification. Scale bar, 100 µm.

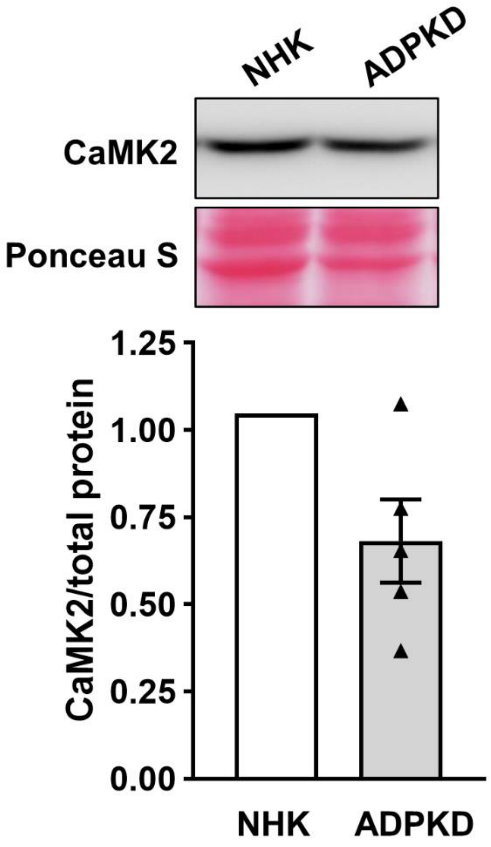

Supplemental Figure S2. CaMK2 expression in normal human kidney (NHK) and ADPKD cells. Representative immunoblots for CaMK2 and Ponceau S staining from lysates of NHK and ADPKD cells grown in culture. Bar graph shows the mean ± SEM for the density of the CaMK4 band normalized to total protein in the entire lane stained by Ponceau S.

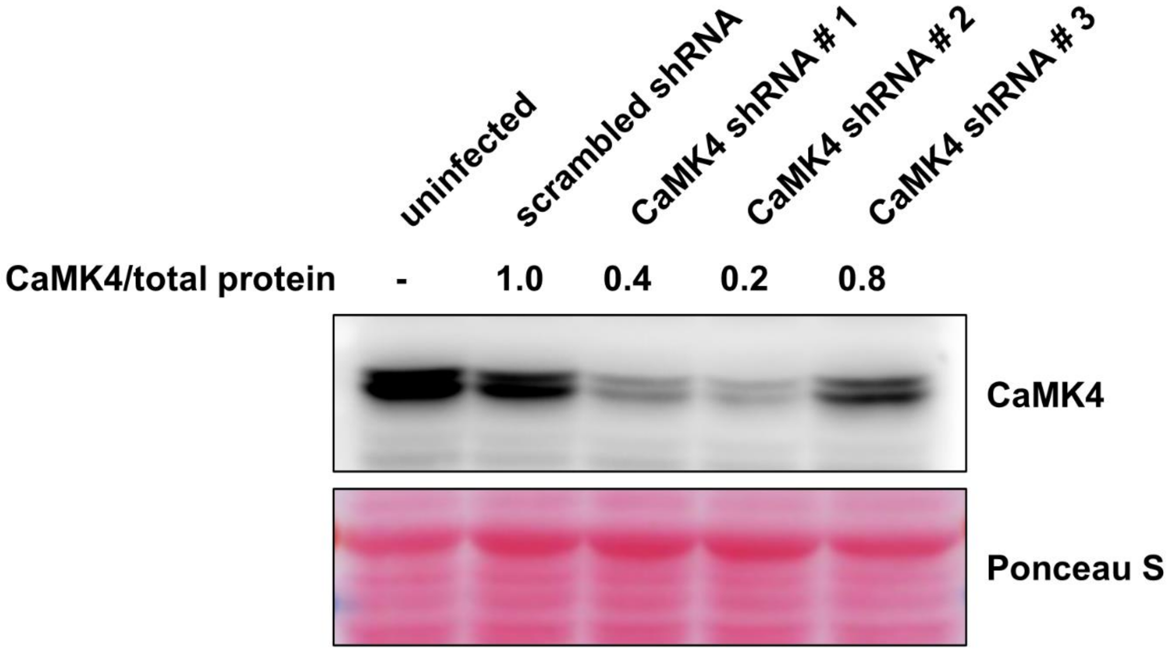

Supplemental Figure S3. CaMK4 knockdown using shRNA in human ADPKD cells. Representative immunoblots for CaMK4 and Ponceau S staining from lysates of ADPKD cells infected with lentivirus carrying a scrambled shRNA or three different CaMK4 shRNA constructs. Numbers above the representative blots are the ratios of CaMK4 intensity and total protein per lane stained by Ponceau S, normalized to the scrambled shRNA band (set to 1.0).

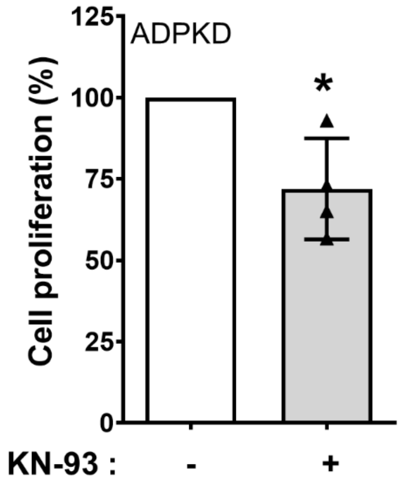

Supplemental Figure S4. Effect of KN-93 on ADPKD cell proliferation. ADPKD cells were treated with 0.01% DMSO (vehicle) or 1 μM KN-93 for 72 hours, cell numbers were determined by a Bio-Rad T20 cell counter. Bars represent mean ± SEM for relative cell proliferation. Cell numbers were normalized to vehicle group (set to 100%). Significant differences were determined by unpaired t-test, *P<0.05, compared with the vehicle group.

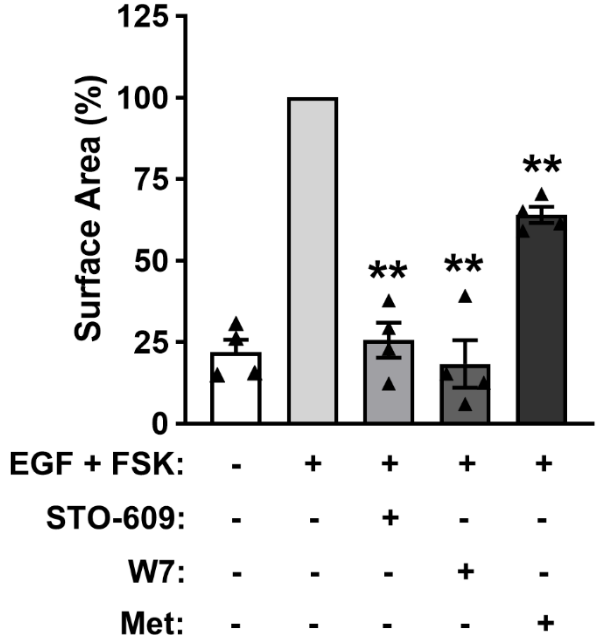

Supplemental Figure S5. Effects of STO-609, W7, and metformin on *in vitro* cyst growth of ADPKD cells. ADPKD cells were seeded within a semi-solid Type I collagen matrix and stimulated with 5 ng/ml EGF + 5 µM FSK for 3 days to initiate cyst formation. EGF and FSK were removed, and the cysts were treated with control media (no agonists), 5 ng/ml EGF + 5 µM FSK alone or with 20 µM STO-609, 10 µM W7 or 1 mM metformin (Met) for approximately 6 days. Media were changed every 2 or 3 days. Bars represent mean ± SEM for total cyst surface area per well of 96-well plates. N = 4 different ADPKD cell preparations. Values were normalized to EGF + FSK group (set to 100%). One-way ANOVA followed by a S-N-K post-test was used to determine significant differences among the groups. ***P*<0.01 compared with EGF+FSK groups.

Supplementary References

Gainullin, V.G., Hopp, K., Ward, C.J., et al. (2015). Polycystin-1 maturation requires polycystin-2 in a dose-dependent manner. J Clin Invest *125*, 607-620.

Pei, Y. (2011). Practical genetics for autosomal dominant polycystic kidney disease. Nephron Clin Pract *118*, c19-30.

Pinto, C.S., Raman, A., Reif, G.A., et al. (2016). Phosphodiesterase Isoform Regulation of Cell Proliferation and Fluid Secretion in Autosomal Dominant Polycystic Kidney Disease. J Am Soc Nephrol *27*, 1124-1134.

Radadiya, P.S., Thornton, M.M., Daniel, E.A., et al. (2021). Quinomycin A reduces cyst progression in polycystic kidney disease. FASEB J *35*, e21533.

Raman, A., Parnell, S.C., Zhang, Y., et al. (2018). Periostin overexpression in collecting ducts accelerates renal cyst growth and fibrosis in polycystic kidney disease. Am J Physiol Renal Physiol *315*, F1695-F1707.

Raman, A., Reif, G.A., Dai, Y., et al. (2017). Integrin-Linked Kinase Signaling Promotes Cyst Growth and Fibrosis in Polycystic Kidney Disease. J Am Soc Nephrol *28*, 2708-2719.

Sharma, M., Reif, G.A., and Wallace, D.P. (2019). In vitro cyst formation of ADPKD cells. Methods Cell Biol *153*, 93-111.

Zhang, Y., Dai, Y., Raman, A., et al. (2020). Overexpression of TGF-beta1 induces renal fibrosis and accelerates the decline in kidney function in polycystic kidney disease. Am J Physiol Renal Physiol *319*, F1135-F1148.
